# Neuroendoscopic Surgery versus External Ventricular Drainage Alone or with Intraventricular Fibrinolysis for Intraventricular Hemorrhage Secondary to Spontaneous Supratentorial Hemorrhage: A Systematic Review and Meta-Analysis 

**DOI:** 10.1371/journal.pone.0080599

**Published:** 2013-11-13

**Authors:** Yuping Li, Hengzhu Zhang, Xiaodong Wang, Lei She, Zhengcun Yan, Nan Zhang, Renfei Du, Kaixuan Yan, Enxi Xu, Lujun Pang

**Affiliations:** 1 Department of Neurosurgery, Clinical Medical College of Yang Zhou University, Yangzhou, Jiangsu Province, China; 2 Department of Neurosurgery, the First People’s Hospital of Zhenjiang, Zhenjiang, Jiangsu Province, China; University of Glasgow, United Kingdom

## Abstract

**Background and Purpose:**

Although neuroendoscopy (NE) has been applied to many cerebral diseases, the effect of NE for intraventricular hemorrhage (IVH) secondary to spontaneous supratentorial hemorrhage remains controversial. The purpose of this study was to analyze the effect of NE compared with external ventricular drainage (EVD) alone or with intraventricular fibrinolysis (IVF) on the management of IVH secondary to spontaneous supratentorial hemorrhage.

**Methodology/ Principal Findings:**

A systematic search of electronic databases (PubMed, EMBASE, OVID, Web of Science, The Cochrane Library, CBM, VIP, CNKI, and Wan Fang database) was performed to identify related studies published from 1970 to 2013. Randomized controlled trials (RCTs) or observational studies (OS) comparing NE with EVD alone or with IVF for the treatment of IVH were included. The quality of the included trials was assessed by Jaded scale and the Newcastle-Ottawa Scale (NOS). RevMan 5.1 software was used to conduct the meta-analysis.

**Results:**

Eleven trials (5 RCTs and 6 ORs) involving 680 patients were included. The odds ratio (OR) showed a statistically significant difference between the NE + EVD and EVD + IVF groups in terms of mortality (OR, 0.31; 95% CI, 0.16-0.59; P=0.0004), effective hematoma evacuation rate (OR, 25.50, 95%CI; 14.30, 45.45; P<0.00001), good functional outcome (GFO) (OR, 4.51; (95%CI, 2.81-7.72; P<0.00001), and the ventriculo-peritoneal (VP) shunt dependence rate (OR, 0.16; 95%CI; 0.06, 0.40; P<0.0001).

**Conclusion:**

Applying neuroendoscopic approach with EVD may be a better management for IVH secondary to spontaneous supratentorial hemorrhage than NE + IVF. However, there is still no concluive evidence regarding the preference of NE vs. EVD alone in the case of IVH, because insufficient data has been published thus far. This study suggests that the NE approach with EVD could become an alternative to EVD + IVF for IVH in the future.

## Introduction

 Intraventricular hemorrhage (IVH) is common disease in neurosurgery, and is mostly secondary to spontaneous intracerebral hemorrhage (ICH), traumatic brain injury (TBI) or aneurysmal and arteriovenous malformation rupture [1]. IVH is a proven risk factor for poor prognosis, and mortality estimates for IVH range from 50% to 80% [2,3]. For IVH secondary to spontaneous supratentorial hemorrhage, the mortality and poor prognosis rate are 72% and 86%, respectively [4]. The outcome is often worsened by development of acute hydrocephalus, mass effect of ventricular blood, the toxicity of intraventricular blood clots, and chronic hydrocephalus. 

 During the past two decades, the medical and surgical management of IVH has remained one of the most difficult challenges for most neurosurgeons. Early treatment of IVH focused on the control of intracranial pressure (ICP). However, it had limited effects on avoiding acute and delayed hydrocephalus. Although the best medical management had been applied, mortality continues to be as high as 50% and the first year survival rate of IVH is only 38% [5]. External ventricular drainage (EVD) is the choice for controlling acute obstructive hydrocephalus, and a systematic analysis confirmed that EVD could significantly decrease the mortality of IVH [6]. Meanwhile, no study has thus far proven that EVD alone could effectively improve the functional outcome of IVH patient and prevent the development of hydrocephalus, which may suggest that there are other risk factors affecting the long term prognosis. The noxious effects of IVH may cause impairment of cerebrospinal fluid circulation, intracranial hypertension, and the development of hydrocephalus [7-9]. Therefore, expeditious evacuation of the intraventricular blood appears to be the only way to reduce mortality and the incidence of hydrocephalus [10]. A multivariate analysis conducted by Steiner T 2006 [11] provided substantial support that faster removal of IVH was an excellent therapeutic target. The aim of intraventricular fibrinolysis (IVF) is aiming to maintain catheter patency and speed up the resolution and drainage of intraventricular blood by applying thrombolytic agents (e.g., rtPA and urokinase). Some case series [12-14] and a recent meta-analysis [15] presented improved survival and functional outcome in IVH patient treated by IVF as compared to EVD alone. However, due to the low quality of the studies, there were not sufficient data to support IVF prevents the development of chronic hydrocephalus in patients with IVH. 

 Currently, early evacuation of IVH could limit the negative effects of ventricular blood clots and prevent delayed hydrocephalus. Several studies [16-19] applied a minimally invasive technique, neuroendoscopy (NE), for fast and complete evacuation of IVH early on, and achieved a good functional outcome and low VP-dependence rate in patients with IVH secondary to hypertensive ICH.

 The purpose of this present study was to evaluate the efficacy and safety of the NE approach compared with EVD alone or with IVF in the treatment of IVH secondary to spontaneous supratentorial hemorrhage. Therefore, we conducted this meta analysis and review the relative literature.

## Materials and Methods

### Literature Search and Study Selection

 Relevant studies were identified by systematic searches of the published articles comparing NE versus EVD alone or with IVF for patients with IVH secondary to spontaneous supratentorial hemorrhage (YP.L and N.Z). The search was not restricted to articles in English and included articles published between January 1966 and April 2013. We searched for relevant studies in the English electronic databases (PubMed, EMBASE, and the Cochrane Library) and Chinese electronic database (CBM, VIP, CNKI, WanFang). The search strategy used both medical subject headings (MeSH) term and keywords searches for intraventricular hemorrhage, IVH, intracerebral hemorrhage, ICH, neuroendoscopy, endoscopy, external ventricular drainage, EVD, ventriculo-peritoneal (VP) shunt, intraventricular fibrinolysis, IVF, which were combined with the Boolean connectors. We looked through grey literature in China through the Chinese Academic Conference Pap (CACP), and we also search the unfinished clinical trials in the Cochrane central registry of controlled trials database to identify relevant journal and reference lists of retrieved articles.

**Figure 1:**
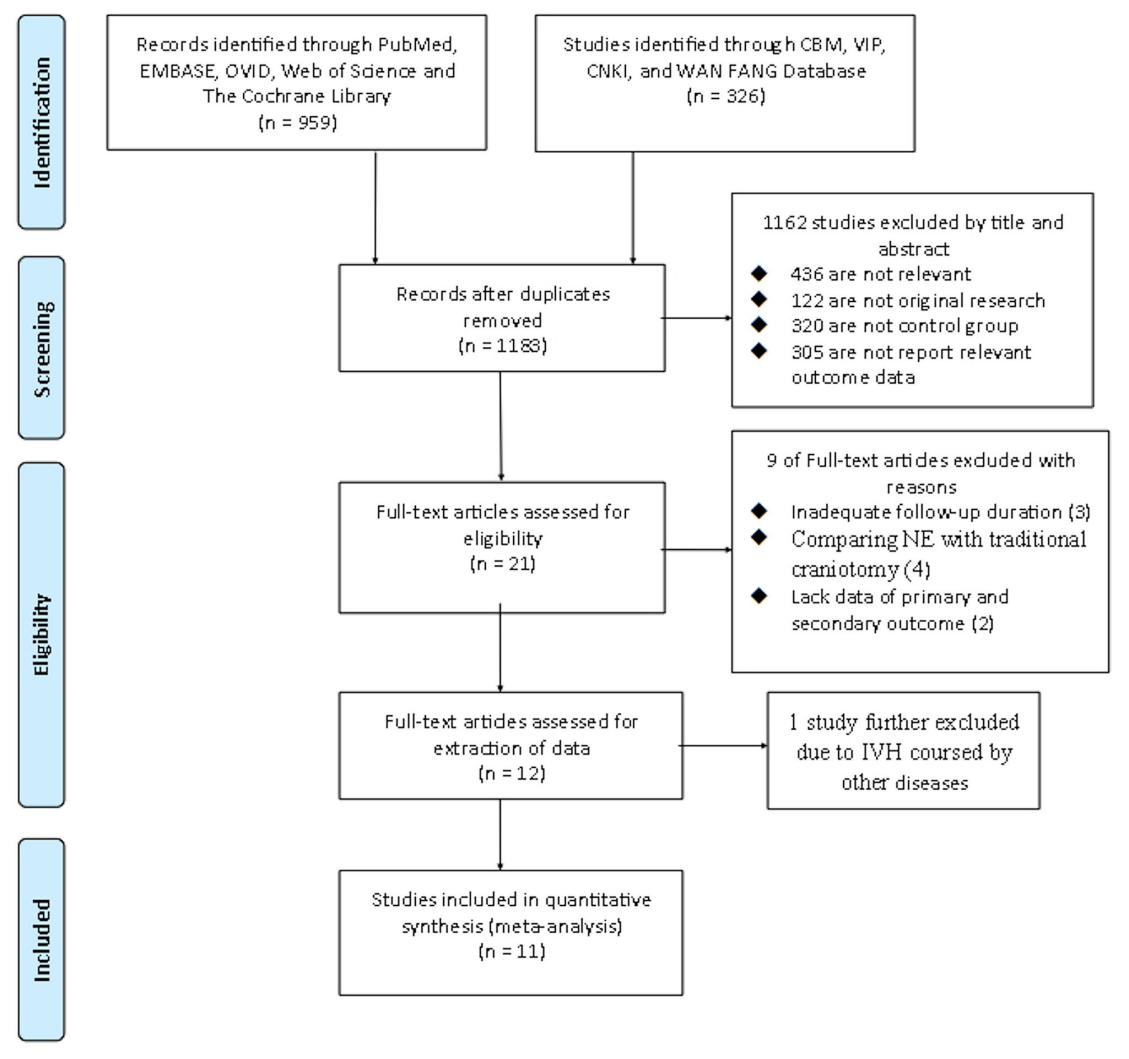
The PRISMA flow chart of the meta-analysis.

 Two independent reviewers (YP.L and N.Z) assessed the literature based on the titles and abstracts to identify potentially relevant articles. Disagreements were resolved through a discussion. Full versions of all relevant articles were obtained and inspected. Literature selection was present in the PRISMA flow chart (Figure 1) according to the PAISMA guidelines [20].

### Inclusion Criteria

When the primary electronic search was completed, a well designed randomized controlled trial (RCT) had not been found. Therefore, we decided to include both small RCTs and prospective or retrospective observational studies (OSs) in this meta-analysis. The following inclusion criteria were used for selecting the potential studies: (1) the study reports results of comparing NE with EVD alone or NE +EVD with EVD + IVF for IVH; (2) the patients were adults; (3) the study reported the outcome measures of the meta-analysis (reported primary or secondary outcomes); and (4) at least 2 months follow-up. 

### Outcome Measures

 The primary outcome was mortality at the end of the follow-up (2 months). Secondary outcomes included the following: (1) effective hematoma evacuation rate, defined as hematoma evacuation rate >60%; (2) good functional outcome (GFO), defined as a patient being able to care for him/herself, corresponding to a modified Rankin Scale (mRS) of 0, 1, 2, or 3, a Glasgow Outcome Scale (GOS) of 4 or 5, or a Activities of Daily Living (ADL) score [21] of 1, 2, or 3; (3) Ventriculo-peritoneal (VP) dependent rate.

### Data Extraction and qualitative assessment

 The relevant data from selected studies were independently extracted by 2 reviewers (YP.L and N.Z). The following pieces of information were extracted: author name, publication year, sample size, study group (mean age, number of patient), GCS on admission, the Graeb score, type of study, surgical procedure, information regarding study quality, follow-up, primary and secondary outcomes. 

 Methodological quality of the including studies was assessed by two observers. The Jaded scale 1996 [22] and the Newcastle-Ottawa Scale (NOS) [23] were introduced to evaluate methodological quality of RCTs and OSs. 

### Statistical Methods

 Meta-analysis was performed using the RevMan software (version 5.1; The Cochrane Collaboration). The odds ratio (OR) with 95% confidence intervals (CIs) was used to assess outcomes of the studies, including mortality rate, effective hematoma evacuation rate, GFO, and VP-dependence rate. Statistical significance was accepted as P value less than 0.05. Because of the small number of studies included in this meta-analysis, I Square value statistics were performed to evaluate heterogeneity between NE and EVD group in each study. The OR was calculated by applying the fixed effect model of random effect model according to I^2^ values which defined 0-25% as low, 25-50% as moderate, 50-75% as high, and > 75% as extreme. Both Begg’s funnel plot and “fail-safe” numbers [24] were performed to assess the publication bias of the literature. The sensitivity analysis was performed in each study and the impact of different interventions was evaluated.

## Results

### Description of the Studies

Figure 1 shows a flow chart of the study selection and inclusion process. The primary search yielded 1285 potentially relevant articles (Figure 1). Of these, 1162 were excluded after reading the title and abstract. Then the full text of the remaining 21 articles was read by 2 independent reviewers (YP.L and HZ.Z). Nine studies were further excluded because of inadequate postoperative follow-up duration (<1 month, 3 articles) and insufficient clinical data (comparing NE with traditional craniotomy, 4 article, or cannot extract the data of primary or secondary outcomes, 2 articles). 

 Twelve articles were identified in this meta-analysis. The Basaldella’s study [17] was excluded because the reported cases of IVH had causes other than spontaneous supratentorial ICH, including ruptured aneurysms, pure IVHs, arteriovenous malformations (AVMs), or posterior fossa hemorrhages. Finally, we included 11 studies [16,18,19,25-32] with a total of 680 IVH patients (Table 1). The sample size of the trials ranged from 18 to 140. Three studies were published in English [16,18,19], and 8 in Chinese [25-32]. These articles were published between 2007 and 2013. Five studies were described as RCT [16,19,26,27,30], and other 6 articles [18,25,28,29,31,32], which lack an optimal randomization method, were included as OS. These 5 RCTs included 388 IVH patients with 191 patients treated through the NE approach (49.2%). The six observational studies included 292 IVH patients, of whom 147 underwent NE (50.3%). One study [29] was prospective, and 5 were retrospective [18,25,28,31,32]. In the included studies, two articles [18,29] applied the NE approach alone, and 9 studies used EVD followed by the NE procedure. We used patients treated with EVD alone or with IVF as the control group. The control group of three studies [18,19,29] was EVD alone, and that of the other 8 studies [16,25-28,30-32] was EVD + IVF. Seven studies clearly described the detail of the fibrinolysis agent and dose of IVF. In 3 studies [18,19,26], the results of GFO were presented in means ± the standard deviationists so that we could not extract the data according to definition of GFO. The other studies clearly presented the following outcome measures: three used GOS, 2 used mRS, and 5 used ADL at 6 months after the operation. The details of the surgical procedure and functional outcome measures are shown in Table 2. Because the studies available utilized different methodologies, we performed two comparisons in our meta-analysis, including NE versus EVD alone and NE + EVD versus EVD + IVF. 

**Table 1:** Characteristics of included studies.

			Initial GCS		Graeb Scale		Age (y)		Cases (M)				
Study	Year	Inclusion criteria	NE	EVD	NE	EVD	NE	EVD	NE	EVD	Outcomes	Side-effect	Followup(m)
Zhang Z16	2007	Any age, < 48 h,	9 (8-12)	6(8-12)	NR	NR	58	58	22(13)	20(12)	Mortality	Rebleeding rate	2
		Diagnosed by CT	13 (<8)	14(<8)							GOS(2 m)	Cerebral infection	
		ICH < 30 ml											
Fuminari K18	2010	Any age, IVH	5.4	7.5	8.9	7.8	58.9	64.3	10(7)	8(6)	Mortality	Rebleeding rate	6
		Acute hydrocephalus									mRS(12 m)	Cerebral infection	
											VP dependent		
											EVD duration		
Chen CC19	2011	Any age, < 48 h	8.54	9.83	6.9	4.54	65.54	62.17	24(NR)	24(NR)	Mortality	Not mentioned	3
		Acute hydrocephalus									VP shunt rate		
		IVH from ICH									GOS(3m)		
TM Song23	2010	Age < 70, < 24 h	9.21	9.27	NR	NR	62.5	61.9	28(15)	25(13)	Evacuation rate	Not mentioned	2
		Diagnosed by CT									GCS(2w and 2m)		
		No trauma history											
M Lang24	2009	20-76 years, < 24 h	22(12-14)	22(12-14)	22(3-5)	22(3-5)	56.3	54.2	80(56)	60(36)	Mortality	Hydrocephalus	6
		IVH caused by ICH	35(10-12)	29(10-12)	35(5-7)	29(5-7)					Evacuation rate		
			23(8-10)	9(8-10)	23(8-10)	9(8-10)					ADL Scale		
											VP shunt rate		
											GCS(2w and 2m)		
HB Duan25	2007	Any age, < 48 h	12(12-14)	23(12-14)	12(3-5)	23(3-5)	53.1	55.8	33(21)	61(40)	Evacuation rates		
		Diagnosed by CT	15(10-12)	29(10-12)	15(5-7)	29(5-7)					VP shunt rate		
		ICH > 30 ml	6(8-10)	9(8-10)	6(8-10)	9(8-10)					GCS(2w and 2m)		
		IVH from ICH									GOS(3m)		
HL Zhang26	2008	Any age, < 6 h	10.3	11.5	7.4	7.1	62.7	61.8	37(22)	33(20)	Mortality	Not mentioned	3
		Pupil mydriasis < 1h									Evacuation rate		
		Hypertension history									GOS(3 m)		
		IVH caused by ICH											
LL Yu27	2012	Any age	All 11.25		All 6.89		55.7	57.3	40(25)	40(27)	Mortality	Not mentioned	6
		Hypertension history									Evacuation rate		
		IVH from ICH									ADL Scale (at 6 mo)		
ZW Lv28	2011	Any age	NR	NR	NR	NR	NR	NR	32(17)	32(18)	Mortality	Cerebral infection	6
		Hypertension history									ADL Scale (6 m)	Hydrocephalus	
		Diagnosed by CT									VP hunt rate		
		ICH <30 ml											
LF Wang29	2011	Any age, < 48 h	4(9-12)	6(9-12)	NR	NR	57.8	59.2	17(11)	22(14)	Mortality	Cerebral infection	6
		Hypertension history	11(7-9)	12(7-9)							ADL Scale (6 m)	Hydrocephalus	
		IVH diagnosed by CT	2(5-6)	12(5-6)							VP hunt rate		
WJ Li30	2013	31-75 years, < 48 h	6(8-14)	7(8-14)	NR	NR	57.5	55.2	21(12)	24(14)	Mortality	Not mentioned	6
		ICH <30 ml	15(<8)	17(<8)							ADL Scale (6 m)		
		IVH caused by ICH									GCS (2w)		

NE: Neuroendoscopy; EVD: External ventricular drainage; IVF: intraventricular fibrinolysis; ICH: intracerebral hemorrhage; VP: Ventriculo-peritonea;l GOS: Glasgow Outcome Scale; mRS: modified Rankin Scale; ADL: Activities of Daily Living; NR: not report;

**Table 2:** Surgical procedure and functional outcome measure of included studies.

		Outcome	GFO(%)			IVF		Fibrinolytic agent				
Study	Design	measures	NE	EVD	Approach	NE	EVD	NE	EVD	Surgical access	VCS	4th ventricular
Zhang Z16	RCT	GOS	13(59)	6(30)	NE + EVD	Y	Y	Urokinase	Urokinase	Frontal	Y	N
								25,000 IU	25,000 IU	Occipital		
								Every 8 hours	Every 8 hours			
Fuminari K18	Retrospective OS	mRS	3(30)	0(0)	NE alone	N	N	--	--	Frontal	N	Y
										Unilateral		
Chen CC19	RCT	GOS	NR	NR	NE + EVD	N	N	--	--	Frontal	N	N
										Occipital		
										Unilateral		
TM Song23	Retrospective OS	GCS	NR	NR	NE + EVD	Y	Y	Urokinase	Urokinase	Frontal	N	N
								20,000 IU	20,000 IU	Unilateral		
								1 time per day	1 time per day			
M Lang24	RCT	ADL	71(88)	38(63)	NE + EVD	Y	Y	Urokinase	Urokinase	Frontal	N	Y
								20,000-40,000 IU	20,000-40,000 IU	Unilateral		
								3 times per day	3 times per day			
HB Duan25	RCT	GCS	NR	NR	NE + EVD	Y	Y	Urokinase	Urokinase	Shortest	Y	N
									20,000 IU			
									2 times per day			
HL Zhang26	Retrospective OS	GOS	27(73)	11(33)	NE + EVD	Y	Y	Urokinase	Urokinase	Frontal	N	Y
								50,000 IU	50,000 IU	Unilateral		
								Every 4 to 8 hours	4 to 8 hours			
LL Yu27	Prospective OS	ADL	35(87)	27(68)	NE alone	N	N	--	--	Frontal	Y	N
										Unilateral		
ZW Lv28	RCT	ADL	27(84)	16(50)	NE + EVD	N	Y	--	Urokinase	Frontal	N	N
									20,000 IU	Occipital		
									Every 4 to 8 hours	Unilateral		
LF Wang29	Retrospective OS	ADL	15(88)	14(63)	NE + EVD	Y	Y	Urokinase	Urokinase	Frontal	N	N
								200,000 IU	200,000 IU	Bilateral		
								2 times per day	2 times per day			
WJ Li30	Retrospective OS	ADL	17(89)	13(76)	NE + EVD	Y	Y	Urokinase	Urokinase	Frontal	NR	N
								200,000 IU	200,000 IU	Unilateral		
								2 times per day	2 times per day			

NE: neuroendocopy; EVD: external ventricular drainage; IVF: intraventricular fibrinolysis; VCS: ventriculocisternostomy; OS: observational study; GOS: Glasgow Outcome Scale; mRS: modified Rankin Scale; ADL: Activities of Daily Living; Y: yes; N: no performed.

### Primary Outcome

#### Mortality of IVH Patients

**Figure 2:**
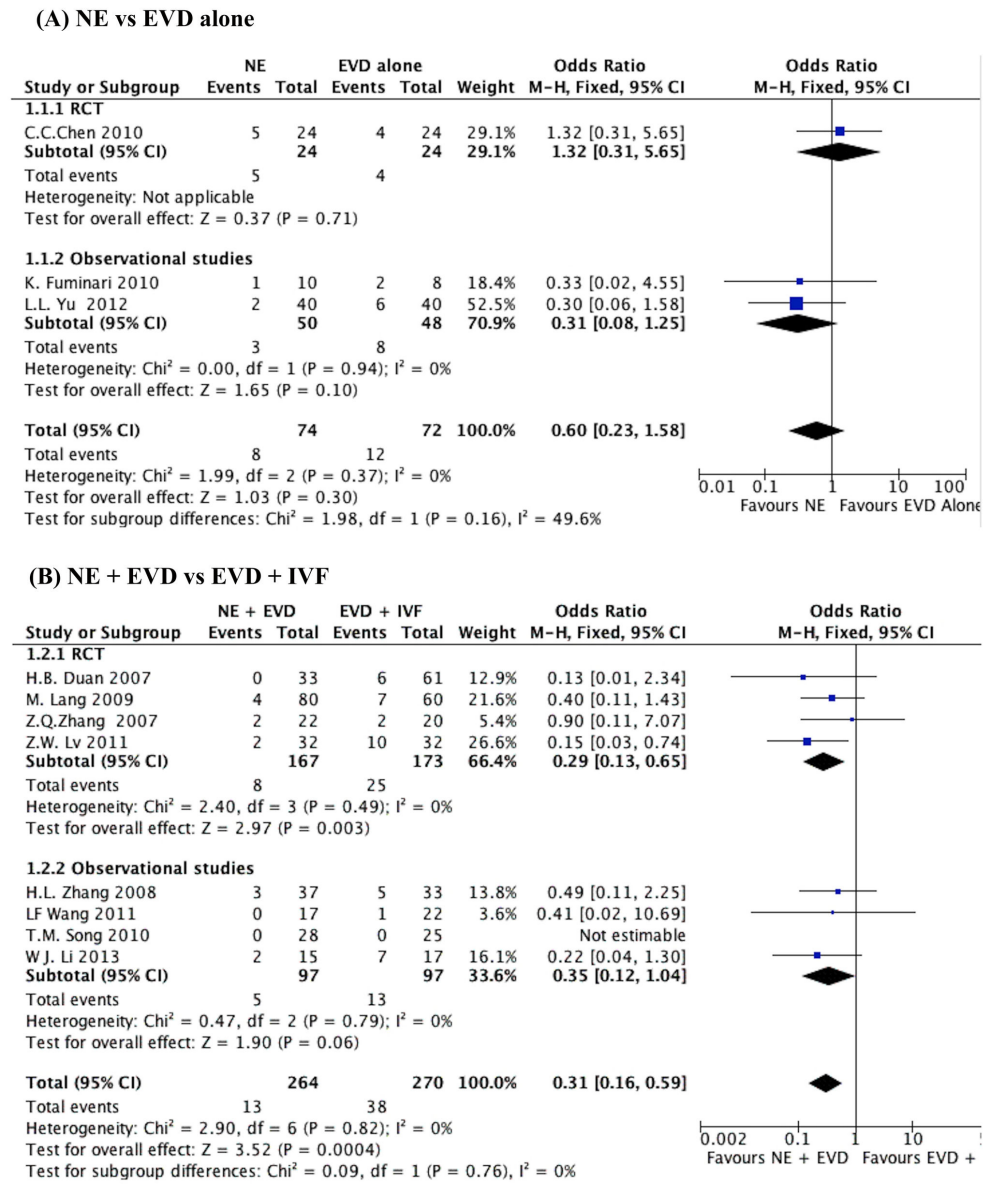
The mortality of IVH patients at the end of the follow-up. (A) NE group versus EVD alone group, (B) NE + EVD group versus EVD +IVF group. (IVH, intraventricular hemorrhage; NE, neuroendoscopy; EVD, external ventricular drainage; IVF, intraventricular fibrinolysis).

All included studies investigated the mortality with 5 RCTs. There were 3 studies [18,19,29] that presented mortality of NE versus EVD alone, including one RCT [19]. When the test of heterogeneity showed no significant differences in each study (I^2^=0), then fixed-effects model was used. A mortality of 14.8% was noted in NE group compared with 16.6% in the EVD alone group. The pooled OR was 0.60 (95% CI, 0.23-1.58; P=0.30), as shown in Figure 2 A. 

Eight studies [16,25-27,30-32] presented mortality of NE + EVD versus EVD + IVF, including 4 RCTs [16,26,27,30]. When the test of heterogeneity had no significant differences in each study (I^2^=0), the fixed-effects model was applied to analyze. A mortality of 4.9% was noted in the NE + EVD group compared with 14.1% in the EVD +IVF group. The overall pooled OR was 0.31 (95% CI, 0.16-0.59; P=0.0004), as shown in Figure 2 B. There was no difference between randomized and observational studies (*P*=0.99).

### Secondary Outcome

#### Effective Hematoma Evacuation Rate

**Figure 3:**
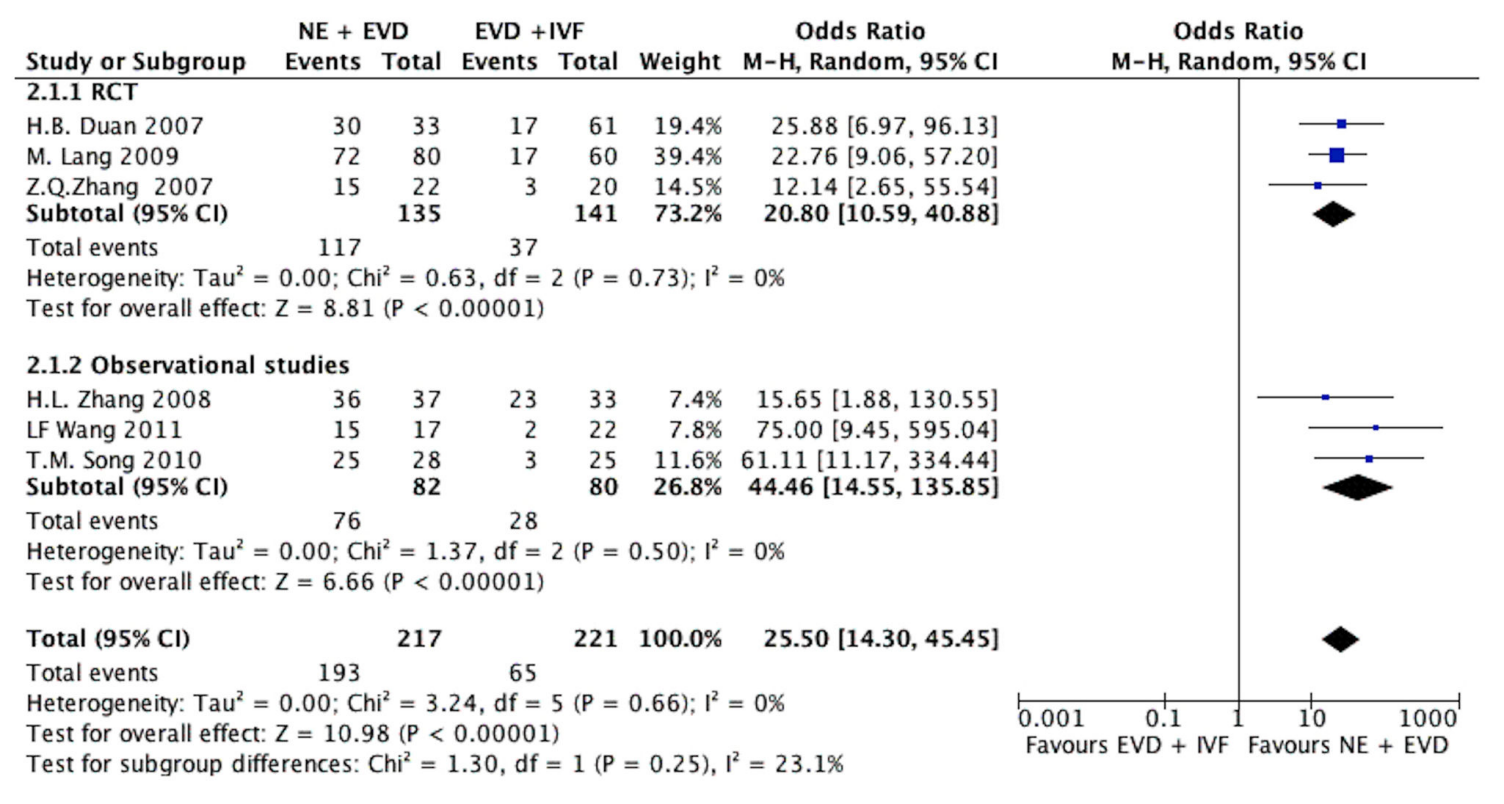
The results of hematoma evacuation rate in IVH patients comparing NE + EVD group and EVD + IVF group. (IVH, intraventricular hemorrhage; NE, neuroendoscopy; EVD, external ventricular drainage; IVF, intraventricular fibrinolysis.)

 Seven studies [16,25-27,29,31] presented data of the effective hematoma evacuation, which was defined as removal of more than 60% of the IVH. One study [29] presented the effective hematoma evacuation rate was 67.5% in the NE group compared to 27.5% in the EVD alone group (*P*<0.05). The other 6 studies compared NE +EVD with EVD +IVF. When statistical heterogeneity among the studies was low (I^2^=0%), the fixed-effects model was adopted. The effective hematoma evacuation rate was 88.9% in the NE + EVD group compared to 29.4% in the EVD +IVF group (P< 0.05). The meta-analysis showed that the overall effective hematoma evacuation rate was statistically higher in the NE + EVD group (OR, 25.50, 95%CI; 14.30, 45.45; P<0.00001) (Figure 3).

#### Good Functional Outcome (GFO)

**Figure 4:**
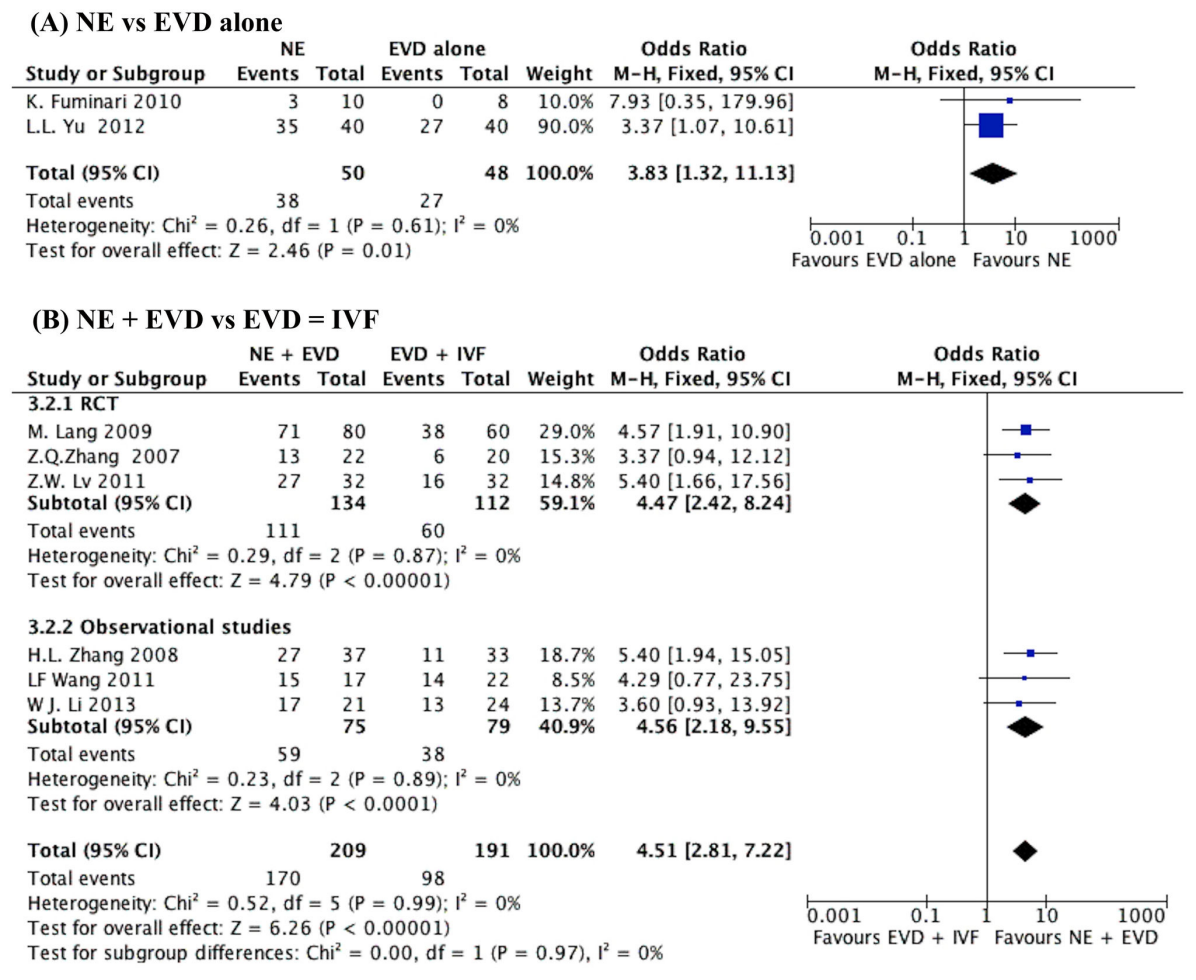
The result of good functional outcome (GFO) in IVH patients. (A) NE group versus EVD alone group, (B) NE + EVD versus EVD + IVF group. (IVH, intraventricular hemorrhage; NE, neuroendoscopy; EVD, external ventricular drainage; IVF, intraventricular fibrinolysis).

 Eight studies [16,18,26,28-32] presented the data of GFO, including 3 RCTs [16,26,30]. Two studies [18,29] compared NE with EVD alone. No statistically significant heterogeneity was observed between studies (I^2^=0%); therefore, the fixed effect model was applied. Meta-analysis showed that the GFO in the NE group was 76%, which was higher than 56% in the EVD alone group (OR, 3.83, 95%CI, 1.32-11.13; P=0.01). (Figure 4 A)

 Six studies reported the GFO comparing NE + EVD with EVD + IVF, including 3 RCTs (Figure 4 B). The test of heterogeneity showed no significant differences in each study (I^2^=0%). The pooled OR was 4.51 (95%CI, 2.81-7.72) with an overall effect of 6.26 (P<0.00001). 

#### The VP-Dependence Rate

Six studies [18,19,26,27,30,31] presented data on the rate of ventriculo-peritoneal shunt (VP shunt) surgery. There were two studies, including one randomized study, comparing NE with EVD alone. In the study by Fuminari [18], none of the IVH patients needed to undergo VP shunt surgery. A study by Chen [19] presented a VP-dependence rate of 47.6% in the NE alone group compared with 90.5% in the EVD alone group (P<0.05). 

**Figure 5:**
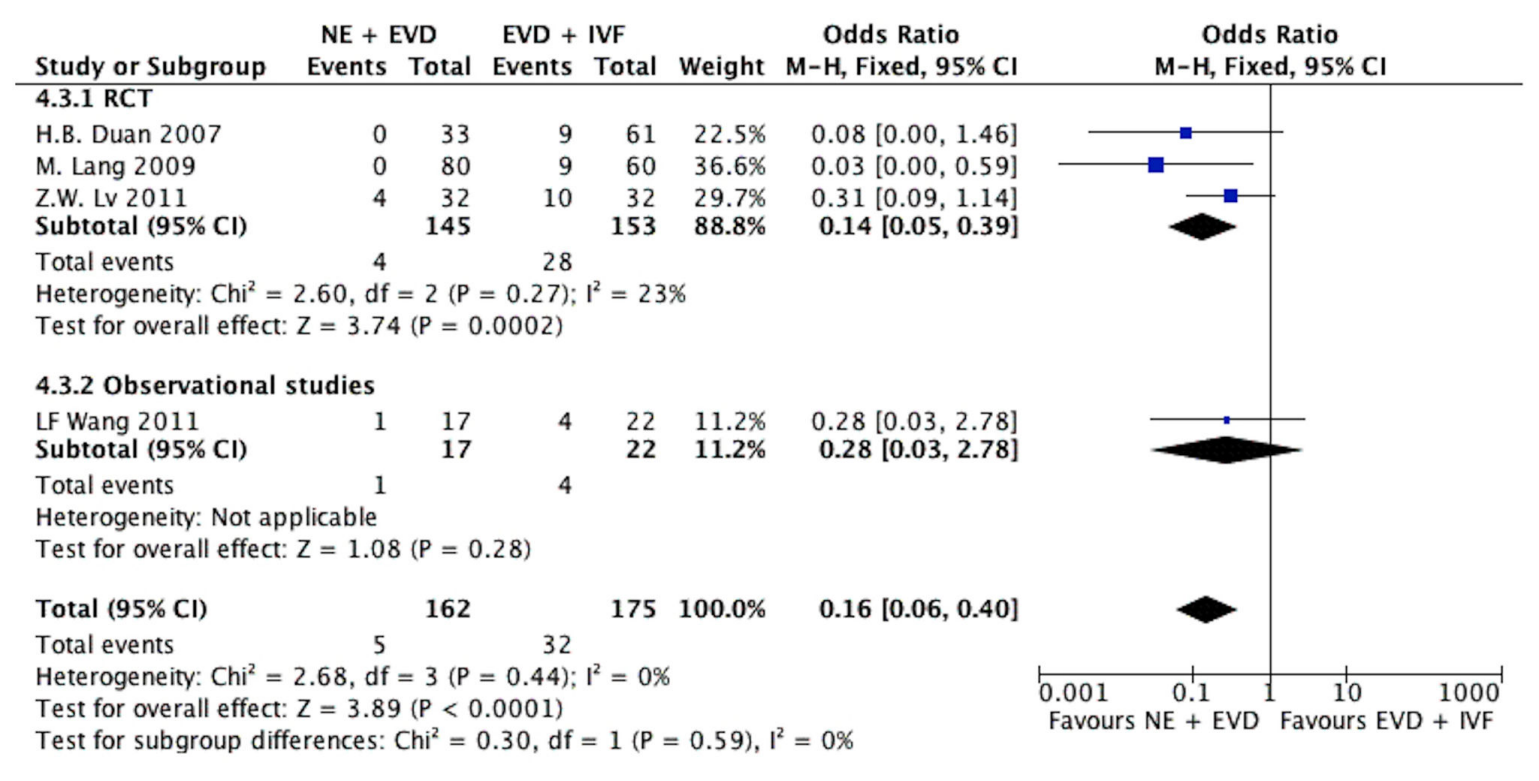
The results of the dependent rate of ventriculo-peritoneal shunt surgery in IVH patients comparing NE + EVD group and EVD + IVF group. (IVH, intraventricular hemorrhage; NE, neuroendoscopy; EVD, external ventricular drainage; IVF, intraventricular fibrinolysis).

 Four studies [26,27,30,31] presented VP-dependence rates comparing NE + EVD with EVD + IVF, including 3 randomized studies (Figure 5). The test of heterogeneity had no significant differences in each study (I^2^=0%); therefore, we used the Peto fixed-effects model. The pooled OR was 0.16 (95%CI; 0.06, 0.40; P<0.0001). There was no difference between the randomized and observational studies (*P*=0.59). 

### Sensitivity Analysis

**Figure 6:**
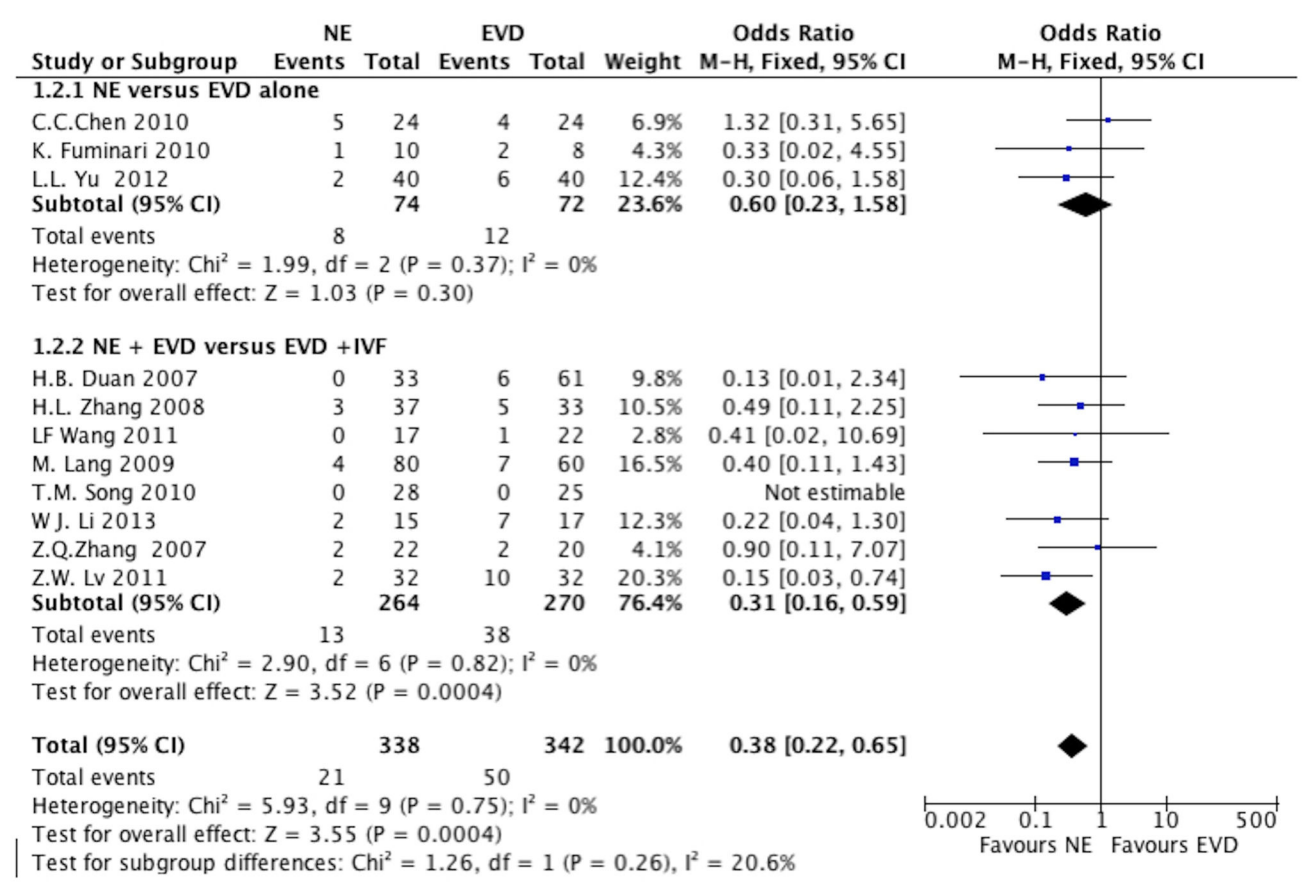
Sensitivity analysis of two intervention comparison (NE versus EVD alone; NE + EVD versus EVD + IVF) on mortality.

 A sensitivity analysis was performed on the studies included in the meta-analysis by deleting each individual data set to assess the influence of the study on the pooled ORs. The results suggested that no individual study significantly affected the pooled ORs. Sensitivity analysis of two different comparisons between NE versus EVD alone and NE + EVD versus EVD + IVF was also performed to identify the effect of IVF on the pooled results, and it showed that the difference between these two comparisons was not significant (P=0.26, test for subgroup differences), thereby indicating that the results are statistically robust (Figure 6).

 Ventriculocisternostomies (VCS) may impact the mortality and prognosis of IVH patients; therefore, the sensitivity analysis was applied determining the effect of VCS between the NE + EVD and EVD + IVF groups. Three studies reported performing VCS in the NE group and those also placed the EVD followed by NE. The results showed no significant difference in mortality, GFO, and VP-dependence rate with P values (test for subgroup differences) of 0.61, 0.98, and 0.95, respectively. The sensitivity analysis revealed no impact of the included studies that had performed VCS on our results (Figure S1-3). 

### Qualitative Assessment and Publication Bias

**Figure 7:**
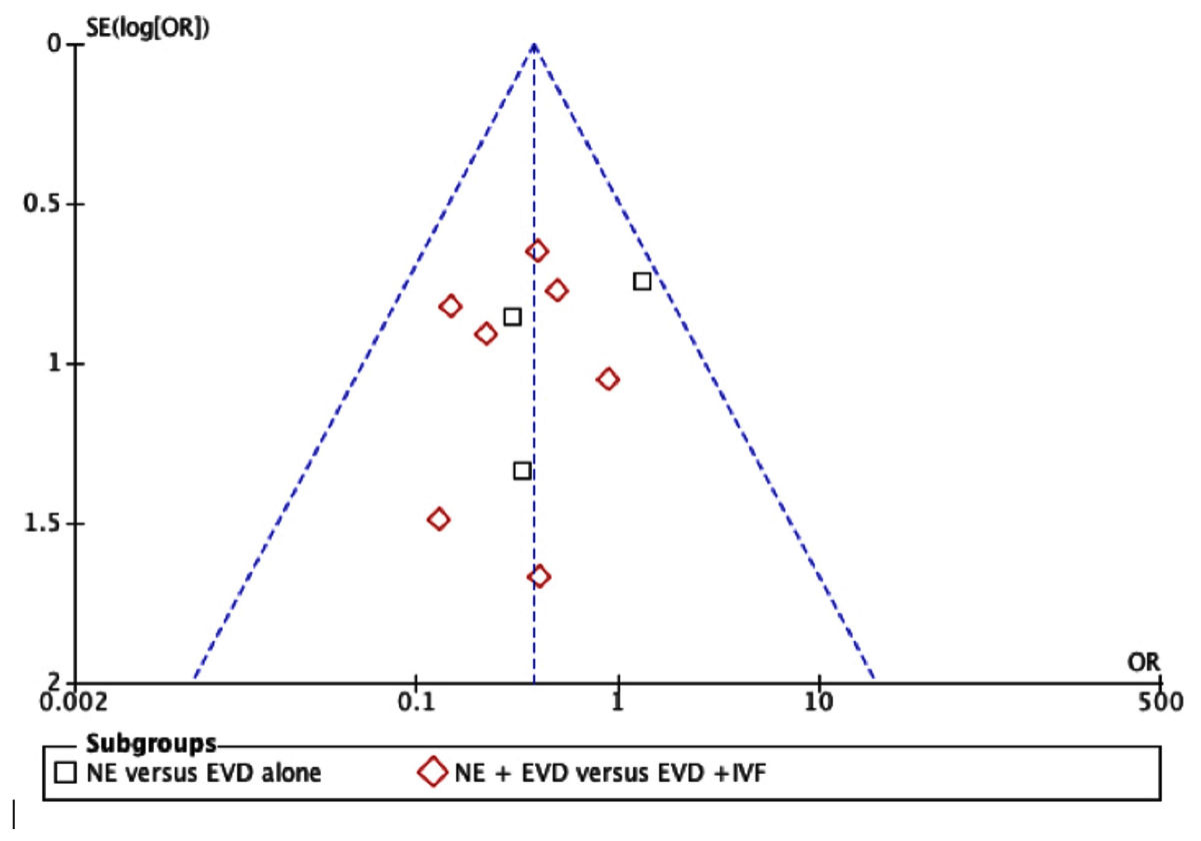
Funnel plot of included studies.

 The quality of the studies included in this meta-analysis is shown in Table 2. It is can be seen from the funnel plot that the publication bias was low regarding mortality (Figure 7), VP-dependence rate and GFO, but moderate regarding hematoma evacuation rate (Figure S4-6 and Table S1). The results of “fail-safe” numbers of included studies on four outcome measures are shown in Table 3.

**Table 3:** Fail-safe numbers of primary and secondary outcome of included studies.

			**Fail-safe numbers**	
**Variable**	**k(number of study)**	**Z**	**Np=0.01**	**Np=0.05**
Mortality	10	12.24743	17.62984	45.77024
Hematoma evacuation rate	7	34.13483	207.6266	426.2193
GFO	8	18.64928	56.06375	121.3113
VP dependent rate	5	12.22171	22.51391	50.53624

The fail-safe numbers are calculated as Np=0.05= (ΣZ/1.64)2-k; Np=0.01 = (ΣZ/2.33)2-k, where k is the number of studies.

The approach presented the potential for unpublished or missing studies to alter our conclusions; a low fail-safe number indicate more complicated publication bias methodologies.

## Discussion

 With a rising interest in minimally invasive techniques, the advancements in neuroimaging have promoted the establishment of modern neuroendoscopy [33]. Endoscopic surgery has many advantages, including minimal invasiveness, high evacuation rate, low incidence of complications, better protection of brain tissue, and less surgery-related injuries [34]. Although NE has been applied to many cerebral diseases, the effect of NE on IVH remains controversial, and it is still not clear whether it can improve the prognosis compared to EVD alone or with IVF. To our knowledge, this study is the first meta-analysis to evaluate the clinical effects of neuroendoscopy in the treatment of IVH. Eleven eligible trials (6 RCTs and 5 OSs) were identified and the pooled result comparing NE + EVD and EVD + IVF in the management of IVH secondary to spontaneous intracerebral hemorrhage showed the superiority of neuroendoscopy on mortality, effective hematoma evacuation rate, GFO and VP-dependence rate. The results indicated that neuroendoscopy with EVD may be superior to EVD + IVF for IVH. However, this study was limited by the small sample of clinical trials (just 5 small RCTs and 6 OSs), and this result still needs further clinical trials for confirmation.

 The high mortality (range from 43% to 83%) and aggressive progression of IVH may relate to the volume of IVH, obstruction of cerebrospinal fluid (CSF) circulation and toxic effect of ventricular blood clots, which could lead to secondary brain damage and acute obstructive hydrocephalus, especially when the third and fourth ventricles are involved [35]. Blood clots in ventricles could obstruct the CSF circulation and cause mass effects. These may lead to obstructive hydrocephalus and secondary brain damage, which is the main reason for neurological deterioration after the first day [36]. An analysis by Hamada of the best available data suggests a 10%-15% absolute benefit might be achieved in IVH subjects, if blood clots are removed from the brain [37]. Therefore, clearing the ventricular hematoma has been shown to dramatically improve CSF circulation and symptoms.

 The debate regarding which surgical intervention should be used to remove the hematoma continues. IVH secondary to spontaneous supratentorial hemorrhage can be treated with the following different surgical interventions: EVD, IVF, and neuroendoscopy. Placement of an EVD was performed to treat acute obstructive hydrocephalus. However, an EVD cannot effectively improve the prognosis of IVH because the catheter is often obstructed by blood clots. A study by Morgan revealed the circadian blood clot dissolution rate was only 10.8% [38]. Furthermore, EVDs also have a high postoperative infection rate, and several studies reported that EVD-related infections have occurred in approximately 10% of IVH patients [39]. 

 In the past two decades, many studies have focused on testing new treatment modalities for IVH, which could result in faster evacuation and drainage of ventricular blood, including IVF and NE. In 2011, a meta-analysis conducted by Gaberel suggested that IVF was probably recommended in IVH secondary to small spontaneous ICH. Recently, an ongoing CLEAR-IVH RCT [15] including 500 patients evaluate the efficacy of the recombinant tissue plasminogen activator (rtPA) in IVF treatment for IVH, and the results will be available in 2015. Therefore, the definitive recommendation remains unanswered. In contrast, neuroendoscopy is an emerging minimal invasive technique, and more widely applied for faster removal of IVH, especially in China. Several small RCTs and observational studies compared these two approaches in treating IVH, and suggested that NE may be as efficient as IVF. Horvath demonstrated that an endoscopic removal of a hematoma offers a more adequate treatment option than EVD placement in patients with IVH [10]. There are other studies [40-42] demonstrating that NE result in high rate of hematoma evacuation (ranging from 83.4-99%). In our study, the mortality in IVH patient showed no statistically significant difference between the NE alone and EVD alone groups. However, the results also showed superiority of NE + EVD compared to EVD + IVF in terms of mortality. Mortality in four different intervention strategies was 4.9% for NE + EVD, 14.1% for EVD + IVF, 14.8% for NE, and 16.6% for EVD alone. Therefore, the placement of an EVD followed by NE maintains the patency of CSF pathways, allowing good control of ICP and faster resumption of normal CSF circulation. These factors may decrease the mortality and improve neurological function. Furthermore, NE could also evacuate most of ventricular blood earlier on, which effectively prevents acute hydrocephalus. In this study, the effective evacuation rate of NE group was 88.9%, which was significantly higher than 29.4% in EVD group (P<0.00001). Thus, the initial goal of IVH management should use the NE approach to evacuate intraventricular blood to reverse the ventricular dilation rapidly and restore normal ICP. 

Chronic hydrocephalus was the most severe complication for IVH and was reported in nearly all recent studies. A study [43] revealed that endoscopic third ventriculostomy was a safe and effective treatment for hydrocephalus related to IVH, and the author reported that 34 IVH patients underwent endoscopic third ventriculostomy, and two of the 34 cases (5.9%) needed VP shunts. Follow-up clinical outcomes indicated that the NE approach could effectively improve the neurological function of patients (P=0.01). To explore proper management of IVH, the NE approach was preferred because of fewer complications and lower VP-dependence rate. Our study showed that VP-dependence rate of the NE + EVD group was significantly lower than the EVD + IVF group (P<0.00001). In the included studies the EVDs were all placed after performance of the NE procedure, 2 of the studies used IVF, and 2 performed VCS, indicating that placement of EVD with IVF followed by NE early on appears to prevent development of chronic hydrocephalus. 

 In terms of prognosis, the following four scales are usually used to evaluate the outcome: the GCS, the GOS, the mRS, and the ADL scale. Recent trials have demonstrated that IVH patients with an initial GCS score of more than 9 had a good recovery [44]. They suggested that the outcomes may depend on the initial GCS. We included two studies that compared GCS between these two interventions. Therefore, we could not analyze the GCS score because we only have two articles and could not extract data from them. In this study, the GFO was applied as a outcome measure to assess neurological functional recovery. We found significant differences in two comparisons. The GFO was 76% in the NE alone group, 56% in EVD alone group, 81.2% in NE + EVD group, and 51.3% in the EVD + IVF group. Thus, use of the NE approach with EVD could potentially improve the prognosis. The satisfactory prognosis through the NE approach may be determined by several benefits as follows: (1) Excellent visual quality in the deep and narrow cerebral ventricular system could enhance the hematoma evacuation rate. (2) Endoscopic surgery through a working channel can significantly reduce the surgery-related injury rate. (3) The incidence of complications (such as the dependence on VP shunts) was lower in the NE group than the EVD alone and EVD + IVF groups. (4) Relative short NICU stays and operative time. 

 Side effects are another problem that needed more consideration. Four of the included studies presented data on complications related to the NE approach. Zhang et al. [16] reported that rebleeding and cerebral infections did not occur in the NE group and that two patients had cerebral infections in the NE with EVD group. Wang reported that infections did not occur in the NE group, while three patients in the EVD + IVF group developed infections. Other included studies did not present any particular complications of the NE procedure. This issue will require further investigation of comparative clinical trials.

### Limitations

 There are several limitations to this study (1). Five RCTs and six OSs were selected in this study, and the sample size in some of them was rather small, which might generate bias of clinical results. In addition, small-volume, observational studies tended to produce more impressive effects than those with large-volume and randomized studies (2). Although the clinical outcomes have shown that neuroendoscopic approach with EVD placement can improve the quality of life for patients, the data on neurological function after 2 years have never been reported (3). There were insufficient data comparing NE versus EVD alone (just three included studies) (4). There is a possibility of publication bias, as suggest by the funnel plot (Figure S4-S6) (5). The random and double-blinded methods are difficult to conduct in the surgical field [45], which are also a limitation that is difficult to eliminate in reality.

## Conclusions

 In summary, this study revealed that applying a neuroendoscopic minimally invasive surgical approach with EVD placement may be a better management of IVH secondary to spontaneous supratentorial hemorrhage than NE + IVF. NE with EVD placement could be an alternative to EVD + IVF for IVH in the future. Although NE with EVD placement for treatment of IVH showed optimistic results in this analysis, further large multicenter randomized controlled trials are still needed to confirm this conclusion. 

## Supporting Information

Checklist S1 **PRISMA 2009 Checklist.**
(DOC)

Table S1 **Qualitative Assessment of included studies.**
(DOC)

Figure S1 **sensitivity analysis of VCS influence the mortality.**
(DOC)

Figure S2 **sensitivity analysis of VCS influence the GFO.**
(DOC)

Figure S3 **sensitivity analysis of VCS influence the VP dependent rate.**
(DOC)

Figure S4 **Funnel plot of hematoma evacuation rate between NE group and EVD + IVF group.**
(DOC)

Figure S5 **Funnel plot of GFO between NE group and EVD + IVF group.**
(DOC)

Figure S6 **Funnel plot of VP dependent rate between NE group and EVD + IVF group.**
(DOC)
