# Supplementary material for: Neuroendoscopic Surgery versus External Ventricular Drainage Alone or with Intraventricular Fibrinolysis for Intraventricular Hemorrhage Secondary to Spontaneous Supratentorial Hemorrhage: A Systematic Review and Meta-Analysis
Source: PLoS One. 2013 Nov 13;8(11):e80599. doi: 10.1371/journal.pone.0080599 (PMC3827437; doi:10.1371/journal.pone.0080599)
Supplement: Table S1 — Qualitative Assessment of included studies. (DOC) [file pone.0080599.s002.doc]

**Table S1 Qualitative assessment** of included studies

|  |  | **Methodological quality** |  |
| --- | --- | --- | --- |
| **Study** | **Characteristics different in two treatment groups** | **NOS score** | **Jadad score** |
| Zhang | NO | - | 3 |
| Fuminari | NO | ★★/★/★★ | - |
| Chen | NO | - | 3 |
| Timu S | NO | ★★/★/★★ | - |
| Ming L | NO | - | 2 |
| Hubing D | NO | - | 2 |
| Heling Z | NO | ★★/★/★★ | - |
| Luli Y | NO | - | 4 |
| Zhengwen L | NO | - | 3 |
| Lifeng W | NO | ★★★/★/★★ | - |
| Wanjun L | NO | ★★/★/★★ | - |

**NE: Neuroendoscopy; EVD: External ventricular drainage; NOS: The Newcastle-Ottawa Scale for Assessing the Quality of Nonrandomized Study in Meta-Analysis; Jadad score: Assessing the quality of reports of randomized clinical trials. NG: No Given.**
