# Supplementary figures and images for: Neuroendoscopic Surgery versus External Ventricular Drainage Alone or with Intraventricular Fibrinolysis for Intraventricular Hemorrhage Secondary to Spontaneous Supratentorial Hemorrhage: A Systematic Review and Meta-Analysis
Source: PLoS One. 2013 Nov 13;8(11):e80599. doi: 10.1371/journal.pone.0080599 (PMC3827437; doi:10.1371/journal.pone.0080599)

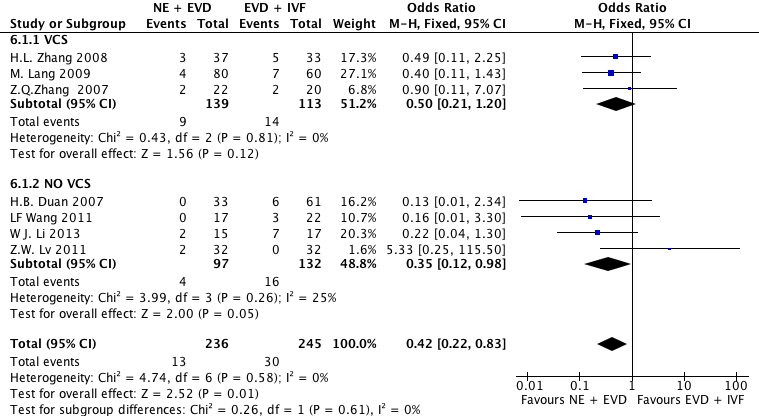


Figure S1 sensitivity analysis of VCS influence the mortality

Supplement: Figure S1 — sensitivity analysis of VCS influence the mortality. (DOC) [file pone.0080599.s003.doc]

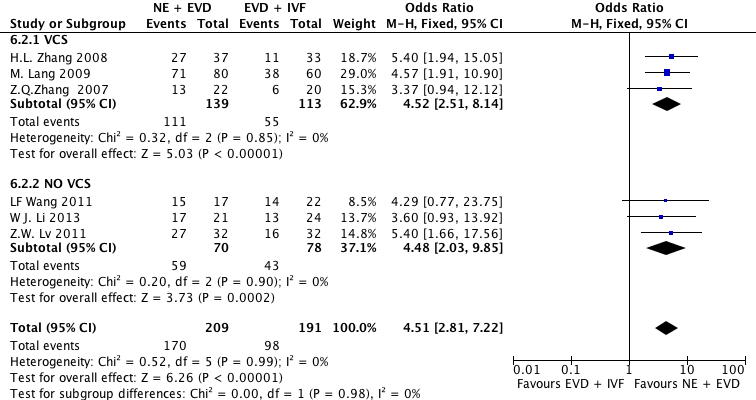


Figure S2 sensitivity analysis of VCS influence the GFO

Supplement: Figure S2 — sensitivity analysis of VCS influence the GFO. (DOC) [file pone.0080599.s004.doc]

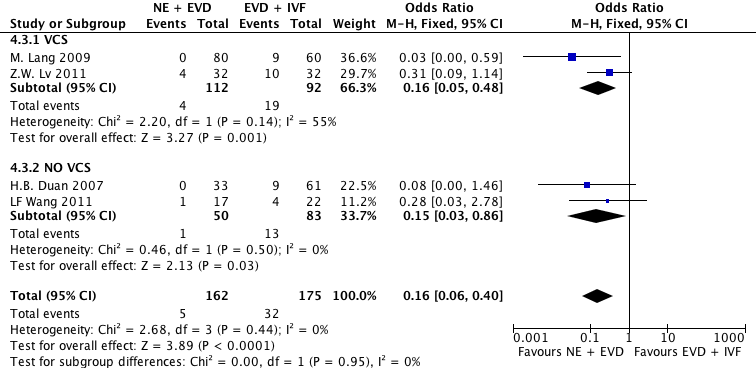


Figure S3 sensitivity analysis of VCS influence the VP dependent rate

Supplement: Figure S3 — sensitivity analysis of VCS influence the VP dependent rate. (DOC) [file pone.0080599.s005.doc]

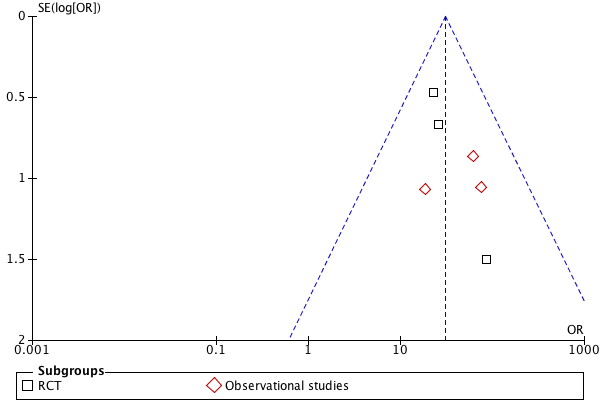


Figure S4: Funnel plot of hematoma evacuation rate between NE group and EVD + IVF group

Supplement: Figure S4 — Funnel plot of hematoma evacuation rate between NE group and EVD + IVF group. (DOC) [file pone.0080599.s006.doc]

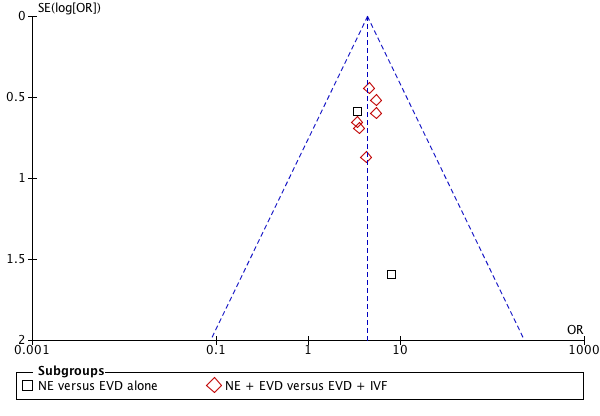


Figure S5: Funnel plot of GFO between NE group and EVD + IVF group

Supplement: Figure S5 — Funnel plot of GFO between NE group and EVD + IVF group. (DOC) [file pone.0080599.s007.doc]

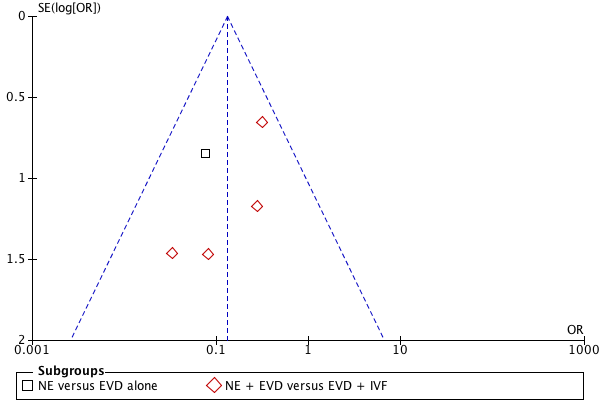


Figure S6: Funnel plot of VP dependent rate between NE group and EVD + IVF group

Supplement: Figure S6 — Funnel plot of VP dependent rate between NE group and EVD + IVF group. (DOC) [file pone.0080599.s008.doc]
